# Scalable Electrophysiological Investigation of iPS Cell-Derived Cardiomyocytes Obtained by a Lentiviral Purification Strategy

**DOI:** 10.3390/jcm4010102

**Published:** 2015-01-07

**Authors:** Stephanie Friedrichs, Daniela Malan, Yvonne Voss, Philipp Sasse

**Affiliations:** 1 Institute of Physiology I, Life and Brain Center, University of Bonn, Bonn 53127, Germany; E-Mails: sfriedri@uni-bonn.de (S.F.); dmalan@uni-bonn.de (D.M.); voss@chemie.uni-siegen.de (Y.V.); 2 Physical Chemistry I, University of Siegen, Siegen 57076, Germany

**Keywords:** induced pluripotent stem cells, cardiomyocytes, purification, long QT syndrome, planar patch clamp, microelectrode array

## Abstract

Disease-specific induced pluripotent stem (iPS) cells can be generated from patients and differentiated into functional cardiomyocytes for characterization of the disease and for drug screening. In order to obtain pure cardiomyocytes for automated electrophysiological investigation, we here report a novel non-clonal purification strategy by using lentiviral gene transfer of a puromycin resistance gene under the control of a cardiac-specific promoter. We have applied this method to our previous reported wild-type and long QT syndrome 3 (LQTS 3)-specific mouse iPS cells and obtained a pure cardiomyocyte population. These cells were investigated by action potential analysis with manual and automatic planar patch clamp technologies, as well as by recording extracellular field potentials using a microelectrode array system. Action potentials and field potentials showed the characteristic prolongation at low heart rates in LQTS 3-specific, but not in wild-type iPS cell-derived cardiomyocytes. Hence, LQTS 3-specific cardiomyocytes can be purified from iPS cells with a lentiviral strategy, maintain the hallmarks of the LQTS 3 disease and can be used for automated electrophysiological characterization and drug screening.

## 1. Introduction

Long QT syndrome (LQTS) is an inherited cardiac disease caused by mutations of cardiac ion channels or accessory subunits, which leads to the loss of function of repolarizing currents or the gain of function of depolarizing currents. Clinically, this disease is characterized by abnormal prolonged QT intervals in the ECG, and the patients affected can develop Torsades de Pointes ventricular tachycardia, which causes syncope and sudden cardiac death [1]. One of the most common LQTS gain of function mutations in humans is the deletion of three amino acids (∆KPQ) in the α-subunit of the cardiac sodium channel (SCN5A) [2], which is classified as LQTS Type 3 (LQTS 3). This mutation results in faster recovery from inactivation of the sodium current and enhanced late sodium currents, which both lead to prolonged action potentials (APs) and early afterdepolarizations (EADs). Because the impact of this mutation is strongest at a low heart rate, lethal cardiac events mostly occur at rest or during sleep [1].

In the past, LQTSs were studied on heterologous expression systems that lack the typical cell biological and physiological features of cardiomyocytes and that do not generate APs [3,4]. Recently, we have shown that LQTS 3-specific cardiomyocytes can be generated from mouse iPS cells carrying the human ∆KPQ mutation and recapitulated the disease-specific biophysical effects of the mutation, as well as prolonged APs and EADs at low heart rates [5]. Furthermore, other groups have successfully generated iPS cells from LQTS 1, 2 and 3 patients, and the cardiomyocytes differentiated from these cells recapitulated the typical characteristics of the respective disease [6,7,8,9,10,11]. Therefore, human iPS cell-derived cardiomyocytes are a great advance for the understanding of LQTS, especially because “real” cardiomyocytes provide a model that is close to the patient’s heart cells.

The unlimited proliferation of personalized iPS cells and the differentiation into cardiomyocytes would allow disease- or even patient-specific drug testing. In order to find new drugs to treat LQTS, pharmaceutical compound libraries have to be screened with scalable automatic assays. Potential automatic electrophysiological screening methods are planar patch clamp systems [12] or microelectrode array technologies [13]. One big challenge for all automated assays is the generation of a pure cardiac population, because during iPS cell differentiation, also non-cardiomyocytes are generated.

To date, several purification methods have been used to enrich cardiac cells. Fluorescence-activated cell sorting of cardiomyocytes with cardiac-specific GFP expression or after labeling with mitochondrial dyes can be used, but these methods result only in low amounts of pure cardiac cells and are difficult to scale up [14,15]. Better yields are achieved with scalable antibiotic selection of cardiomyocytes, which express a resistance gene under a cardiac-specific promoter [16,17]. For antibiotic selection, cells must be genetically modified, and here, we report a highly efficient and straightforward lentiviral gene transfer for the selection of cardiomyocytes by an antibiotic resistance gene without a time-consuming screening of individual clones. We have applied this method to obtain pure populations of cardiomyocytes from LQTS 3-specific iPS cells with the human ∆KPQ mutation and wild-type controls. Furthermore, we proved that purified cardiomyocytes showed the typical features of LQTS 3 in manual patch clamp, automatic planar patch clamp and scalable microelectrode array recording technologies.

## 2. Experimental Section

### 2.1. Generation of the Lentiviral αPaG-RexNeo Plasmid and Lentivirus Production

The lentiviral αPaG-RexNeo plasmid is based on a pRRLSIN lentiviral backbone from pRRLSIN.cPPT.PGK-GFP.WPRE (kindly provided by Didier Trono through Addgene #12252). Multiple cloning steps according to standard procedures were used to create an insert containing a short version of the cardiac-specific alpha myosin heavy chain (α-MHC) promoter, a puromycin resistance gene, the green fluorescence protein (GFP) and a fragment with the Rex-1 promoter driving a neomycin resistance gene. The short α-MHC promoter was excised from the α-MHC-pBK plasmid (kindly provided by Jeffrey Robbins) and contained 1745 bp from the 3′ part of the full α-MHC promoter. Parallel expression of puromycin and GFP was achieved by the introduction of the 2A self-cleaving peptide sequence (APVKQTLNFDLLKLAGDVESNPGP) [18] that was generated by annealing and in-frame ligation of appropriate oligonucleotides (MWG-Biotech, Ebersberg, Germany). The Rex-1-neomycin sequence was cut from the α-MHC-puro Rex-neo plasmid (kindly provided by Mark Mercola through Addgene #21230). Successful cloning was confirmed by restriction enzyme digestion and DNA sequencing (MWG-Biotech, Ebersberg, Germany). All enzymes for cloning were from Life Technologies (Darmstadt, Germany) and Thermo Scientific Fermentas. For the preparation of lentivirus, 40 μg of the αPaG-RexNeo plasmid, 8.5 μg of the pMD2.G plasmid (for the VSV-G envelope, Addgene #12259), 16 μg of the pMDLg/pRRE plasmid (for Gag/Pol expression, Addgene #12251) and 7 μg of the pRSV-Rev plasmid (for Rev expression, Addgene #12253, all kindly provided by Didier Trono through Addgene), were cotransfected into 7 × 10^6^ HEK293FT cells (ATCC) in a T75 culture flask, as previously described [19]. After 24 h, the medium was changed with fresh HEK cell medium that consisted of Dulbecco’s Modified Eagle Medium (DMEM), 15% fetal calf serum (FCS), 0.1 mmol/L MEM nonessential amino acids, 0.1 mmol/L 2-mercaptoethanol, 100 U/mL penicillin and 100 mg/mL streptomycin (all from Invitrogen/Bernardi). Virus-containing supernatants were collected at Days 3 and 4 after transfection, passed through a 0.45-μm filter (Sigma-Aldrich, Taufkirchen, Germany) and concentrated by ultracentrifugation at 19.400 rpm for 2 h at 17 °C using an Optima L-90K ultracentrifuge with an SW 32 Ti rotor (Beckman Coulter, Krefeld, Germany). The pellet was resuspended in 50 μL HBSS without Ca^2+^ and Mg^2+^ (Life Technologies, Darmstadt, Germany) and stored frozen at −80 °C.

### 2.2. Cell Culture and Lentiviral Gene Transfer of iPS Cells

The iPS cells were cultured as reported before [5] on irradiated mouse embryonic feeder (MEF) layers (PMEF-NL; Millipore, Schwalbach, Germany) in iPS cell medium containing DMEM, 15% FCS, 0.1 mmol/L nonessential amino acids, 0.1 mmol/L 2-mercaptoethanol, 100 U/mL penicillin, 100 mg/mL streptomycin (all from Invitrogen/Life Technologies), 1000 U/mL leukemia inhibitory factor (Chemicon/Millipore), 3 μmol/L CHIR99021 and 1 μmol/L PD184352 (Axon Medchem, Groningen, The Netherlands). Every 2 to 3 days, iPS cells were passaged and seeded at a density of 0.1 to 0.2 × 10^6^ cells in a T75 culture flask.

For gene transfer of αPaG-RexNeo, 0.2 × 10^6^ iPS cells were plated on a T25 culture flask on irradiated MEFs, and 24 h later, the αPaG-RexNeo lentivirus from the production described in section 2.1 was added in 5 mL of iPS cell medium in the presence of 6 μg/mL protamine sulfate (Sigma-Aldrich) to enhance infection. The next day, fresh iPS cell medium was applied, and the selection of iPS cells with lentivirus integration was initiated 24 h to 48 h later by the addition of 300 μg/mL neomycin (G418, Invitrogen/Life Technologies). The genetically-engineered wild-type and Scn5a∆/+ iPS cells were further cultivated and passaged in iPS cell medium in the presence of 300 μg/mL neomycin to avoid lentivirus silencing.

### 2.3. Differentiation of iPS Cells and Purification of Cardiomyocytes

Cardiomyocyte differentiation was induced using embryoid body (EB) formation with the hanging drop method in combination with a suspension protocol, as previously described [16]. Briefly, EBs were generated by aggregation of 400 cells in 20 μL differentiation medium for 2 days and subsequently cultured in suspension in 10-cm bacteriological dishes on a horizontal shaker in differentiation medium containing Iscove’s Modified Dulbecco’s Medium, 20% FCS, 0.1 mmol/L MEM nonessential amino acids, 0.1 mmol/L 2-mercaptoethanol, 100 U/mL penicillin, 100 mg/mL streptomycin (all from Invitrogen/Life Technologies). EBs started to beat at day 10 to 12 of differentiation and 10 μg/mL puromycin (Sigma-Aldrich) was added at that time point to initiate the selection of cardiomyocytes. One day later, EBs were pooled, washed with PBS and dissociated with 1 mg/mL collagenase B (Roche Diagnostics, Mannheim, Germany) in 1.6 mL in a 50-mL falcon tube for 60 min at 37 °C under shaking condition. The enzymatic reaction was stopped with the addition of 30 mL of differentiation medium. In order to avoid a centrifugation step that was found to be lethal for the freshly-dissociated cardiomyocytes, a subsequent passive sedimentation step was performed for 60 min in the incubator. The supernatant was removed except ~10 mL, in which the cardiomyocytes were resuspended and collected. For further selection and cultivation, cells were seeded on 0.01% fibronectin-coated (Sigma-Aldrich) 10-cm cell culture dishes in differentiation medium supplemented with 2.5 to 5 μg/mL puromycin. To obtain a more mature stage for electrophysiological analysis, single purified cardiomyocytes were kept in culture for an additional 6 to 10 days, because we have shown that longer differentiation leads to more cells with functional Na^+^ currents [5].

### 2.4. Immunocytochemistry

For immunostainings, cells were fixed with 4% paraformaldehyde for 30 min, permeabilized with 0.2% Triton X-100 for 10 min (both from Sigma-Aldrich) and blocked with 5% donkey or goat serum for 30 min (Jackson ImmunoResearch, Suffolk, England). The primary antibodies were diluted in 0.5% donkey or goat serum, and cells were incubated for 2 h. Colonies of iPS cells were stained against Oct3/4 (rabbit, 1:100; Santa Cruz Biotechnology, Heidelberg, Germany) and SSEA1 (mouse, 1:80; Developmental Studies Hybridoma Bank, Iowa, USA). Single cardiomyocytes were stained against α-actinin (mouse, 1:400; Sigma-Aldrich) and the cardiac Na^+^ channel (Nav1.5, rabbit, 1:400; Alomone Labs, Jerusalem, Israel). The appropriate fluorescence-conjugated secondary antibodies, donkey anti-mouse Cy2-labeled, donkey anti-rabbit Cy3-labeled (both 1:400; Jackson ImmunoResearch) and goat anti-mouse Alexa647-labeled (1:500; Invitrogen/Life Technologies), were diluted in 1 μg/mL of Hoechst 33342 (Sigma-Aldrich) and applied for 1 h. Samples were embedded in polyvinyl alcohol mounting medium (FLUKA; Sigma-Aldrich) and analyzed using an AxioObserver Z1 microscope equipped with an ApoTome optical sectioning device and the AxioVision software (Zeiss, Jena, Germany).

To analyze the purity of iPS cell-derived cardiomyocytes, purified cells at Days 13 to 15 of differentiation from 4 to 5 independent biological replicates were stained against α-actinin. The ratio of α-actinin-positive cells to the total cell number analyzed by nucleus labeling was quantified from large overview pictures that were acquired with the MosaiX function of the AxioVision software (Zeiss).

### 2.5. Conventional Manual Patch Clamp Analysis

Purified wild-type and Scn5a∆/+ cardiomyocytes were dissociated and replated for 48 to 72 h at low densities on fibronectin-coated (0.01%) coverslips. Patch clamp experiments were performed after 48 to 72 h using an EPC10 amplifier (HEKA Elektronik, Lambrecht, Germany) in the whole cell configuration and the current clamp mode, as reported earlier [5], with continuous superfusion with extracellular solution at 37 °C containing (in mmol/L) 140 NaCl, 5.4 KCl, 1.8 CaCl_2_, 1.2 MgCl_2_, 10 Hepes and 10 glucose, pH 7.4 (NaOH), and an internal solution containing (in mmol/L) 50 KCl, 80 K-aspartate, 1 MgCl_2_, 3 MgATP, 10 EGTA and 10 HEPES, pH 7.2 (KOH) (all from Sigma-Aldrich). APs were elicited by 2.5 ms-long current injections, and the strength of the pulse was increased stepwise until a stable action potential with a peak over the 0 mV line was reached. The stimulation frequency and amplitude was controlled by an external stimulator (Model 2100, A–M Systems) attached to the EPC10 amplifier.

### 2.6. Automated Planar Patch Clamp Analysis

For automated planar patch clamp measurements, single dissociated cardiomyocytes are required in suspension without damage of the cell membrane or transmembrane ion channels. Therefore, purified wild-type and Scn5a∆/+ cardiomyocytes in a 10-cm cell culture dish were washed with 5 mL PBS containing EDTA (2 mM) and stored for 10 min at 4 °C in order to facilitate the detachment of cells by subsequent incubation with 2 mL 0.05% Trypsin in 4 mM EDTA (Gibco/Life Technologies) for 3 to 8 min. Cells were collected in 10 mL of differentiation medium, gently centrifuged for 3 min at 500 rpm, resuspended in 200 to 500 μL external solution and incubated at room temperature for at least 2 h to recover from dissociation. Automated electrophysiological recording was performed with a planar patch clamp robot (Patchliner, Nanion Technologies, Munich, Germany) equipped with an EPC-10 quadro patch clamp amplifier (HEKA Elektronik) for parallel recording of 4 cardiomyocytes in the whole cell configuration. Single-use borosilicate glass chips with medium resistance (1.8 to 3 MΩ, NPC-16, Nanion Technologies) were used for all recordings. The PatchControlHT software (Nanion Technologies) in combination with the PatchMaster software (HEKA Elektronik) was used for cell capture, seal formation, whole-cell access and subsequent recording of voltage ramps, automated determination of AP stimulus thresholds and AP measurements at different stimulation frequencies. The internal solution used contained (in mmol/L) 50 KCl, 60 K-fluoride, 10 NaCl, 20 EGTA and 10 HEPES, pH 7.2 (KOH), and the external solution 140 NaCl, 4 KCl, 2 CaCl_2_, 5 Glucose and 10 HEPES, pH 7.4 (NaOH) (all from Sigma-Aldrich). A seal enhancer solution containing (in mmol/L) 80 NaCl, 3 KCl, 10 MgCl_2_, 35 CaCl_2_ and 10 HEPES (Na^+^ salt), pH 7.4 (HCl) (all from Sigma-Aldrich), was automatically applied to the extracellular channel after cell capture in order to achieve better GΩ-seals and replaced with external solution when the whole cell configuration was established.

In order to identify mature cardiomyocytes, depolarizing voltage ramps (−100 mV to +60 mV in 250 ms) were applied, and the responding current was analyzed to identify the fast spike of Na^+^ currents. APs were recorded in current clamp mode and to avoid spontaneous activity, and to record APs from a stable resting potential, the membrane potential was adjusted to −70 mV by current injection using the low frequency voltage clamp circuit of the amplifier. Before each AP recording, the low frequency voltage clamp was switched off, and the actual current was continuously injected to maintain the resting membrane potential. To determine the current injection threshold for AP generation for each cell individually, an automated macro was programmed and executed. This generated a 2-ms current injection of stepwise (100 pA) increasing intensities and automatically monitored the voltage responses. Leak subtraction was used to subtract the passive capacitive responses to the stimulus. Once the stimulus generates voltage responses with an amplitude of >30 mV above the resting membrane potential, this value was used, and 80 pA was added for safety. Subsequently, APs were automatically evoked and recorded for 30 to 60 s at 0.5 Hz, 1 Hz and 2 Hz by a protocol in the Patchmaster software (HEKA Elektronik).

Data from both conventional and planar patch clamp were acquired with the Patchmaster software and analyzed offline using the Fitmaster (HEKA Elektronik) and the Labchart software (AD Instruments, Oxford, England). The action potential duration at 90% of repolarization (APD90) was analyzed with the peak analysis module of Labchart software (AD Instruments). To quantify the frequency-dependent AP duration, cardiomyocytes were stimulated at different pacing periods (0.5 to 6 s for manual patch clamp and 0.5 to 2 s for automatic patch clamp), and at each period, the average APD90 was determined. For each individual cell, the APD90 values were plotted against the period between stimulation (1/frequency), and a linear regression analysis was used to determine the slope of this relationship.

### 2.7. Microelectrode Array Analysis

For the microelectrode array (MEA) measurements, purified cardiomyocytes from wild-type and Scn5a∆/+ iPS cells were detached with 0.05% Trypsin in 0.5 mM EDTA (Gibco/Life Technologies) for 5 min at 37 °C, centrifuged for 5 min at 1000 rpm and resuspended in differentiation medium. Then, 20,000 to 40,000 cells were plated in each well of a 6-well MEA (60-6wellMEA200/30iR-Ti-tcr, Multi Channel Systems, Reutlingen, Germany) coated with 0.01% fibronectin (Sigma-Aldrich). After 24 to 72 h, the medium was replaced with external solution (see Section 2.5), and field potentials were recorded at a sampling rate of 10 kHz with the MC-Rack software at room temperature (22 °C) and at 37 °C by switching on the TC02 2-channel temperature controller (both from Multi Channel Systems). Triggered field potentials were averaged over 50 s, and the mean of all 9 electrodes in one well was calculated (OriginPro8G, OriginLab) to obtain one averaged field potential for further analysis. The field potential duration was manually measured from the minimum of the sharp negative spike to the following maximum (Figure 6c, right).

### 2.8. Statistics

Data are expressed as the mean ± S.E.M. Statistical tests were performed using appropriate unpaired or paired Student’s *t*-test with Welch’s correction for data with unequal variance using Prism (GraphPad software). A *p*-value of <0.05 was considered significant and is indicated by an asterisk (*) in the figures. Because of high variations in temperature-induced frequency between Scn5aΔ/+ and wild-type cardiomyocytes using MEA recordings (Scn5aΔ/+: high 1.4–1.8 Hz, low 0.7–1.0 Hz; wild-type: high 1.6–4.7 Hz, low 1.0–3.5 Hz), in these experiments, only paired Student’s *t*-tests within individual genotypes were performed (Figure 6d).

## 3. Results

### 3.1. Lentiviral Strategy for Purification of iPS Cell-Derived Cardiomyocytes

**Figure 1:**
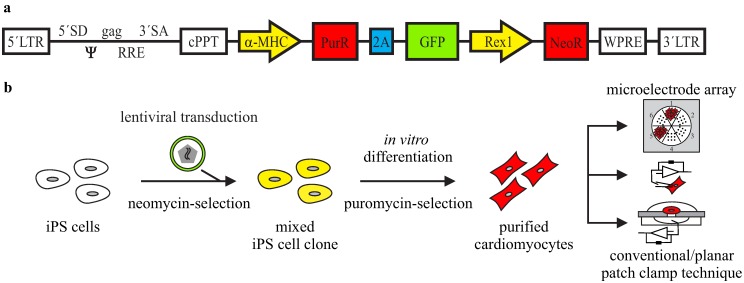

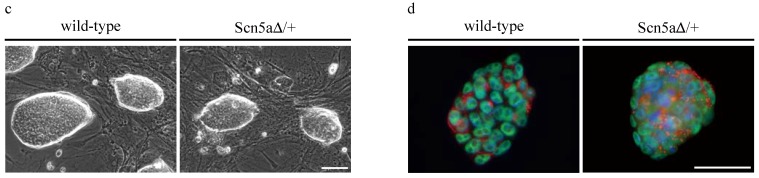
Lentiviral strategy for the purification of iPS cell-derived cardiomyocytes. (**a**) The lentiviral construct contains a puromycin resistance gene (*PurR*) and a GFP reporter gene separated by a 2A self-cleaving peptide sequence (2A) under the control of the cardiac α-MHC promoter, as well as a neomycin resistance gene (*NeoR*) under the control of the Rex1 promoter; (**b**) strategy of lentivirus gene transfer into iPS cells and purification of cardiomyocytes for electrophysiological analysis; (**c**,**d**) after lentiviral gene transfer and selection, wild-type and Scn5a∆/+ iPS cell lines maintained the characteristic embryonic stem cell-like morphology (**c**) and expressed the embryonic stem cell-specific markers, Oct3/4 (**d**, green) and SSEA1 (**d**, red). Nuclei are shown in blue. Scale bars: 50 μm.

In order to obtain a pure cardiomyocyte population from iPS cell differentiation, we have modified a previously-reported antibiotic resistance strategy [16] and used high efficiency lentiviral gene transfer [17]. Therefore, we have generated a lentiviral plasmid (αPaG-RexNeo) for the expression of a puromycin resistance gene and the green fluorescent protein (GFP) reporter gene under the control of a short (1.7 kb) version of the cardiac-specific alpha myosin heavy chain (α-MHC) promoter (Figure 1a). In addition, the plasmid contained a fragment with a neomycin resistance gene expressed under the control of the pluripotency promoter Rex-1 [20]. After infection of cells with this lentiviral plasmid, undifferentiated stem cells with stable integration of the lentivirus can be selected by cultivation in the presence of neomycin [17]. Upon differentiation, cardiomyocytes can be purified by puromycin application and used for electrophysiological investigations (Figure 1b). To test this strategy for the investigation of a clinically relevant cardiac disease, we have purified LQTS 3-specific cardiomyocytes from previously-reported Scn5a∆/+ iPS cells [5] with the human ∆KPQ mutation in the cardiac sodium channel.

Therefore, a monolayer of undifferentiated Scn5a∆/+ and wild-type iPS cells were infected with the αPaG-RexNeo lentivirus and further kept under neomycin selection for the isolation of cells with a stable integration. The surviving iPS cells were collected and pooled for each genotype. Although this non-clonal strategy results in a mixture of individual cell clones with uncontrolled variations in the number and location of lentiviral integrations, it does not require the very laborious picking and characterization of several individual clones. Importantly, after αPaG-RexNeo gene transfer and selection, we found that both wild‑type and LQT 3-specific iPS cells maintained their characteristic embryonic stem cell morphology (Figure 1c) and expressed the stem cell-specific markers Oct3/4 and SSEA1 (Figure 1d).

### 3.2. Purification of αPaG-RexNeo iPS Cell-Derived Cardiomyocytes

**Figure 2:**
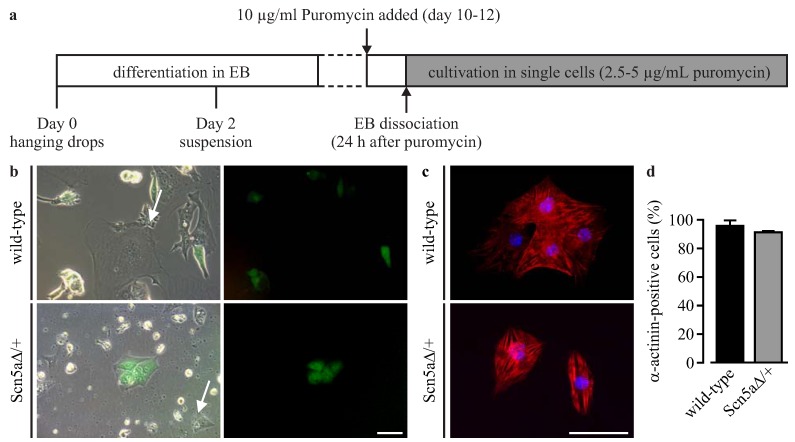
Purification of iPS cell-derived cardiomyocytes. (**a**) The cardiac differentiation protocol used in this study; (**b**) single dissociated cardiomyocytes were GFP-positive and beating, but some non-contracting and GFP-negative cells remained (arrows) after the puromycin selection for 24 h; (**c**) after further purification of single cells, mostly α-actinin-positive cardiomyocytes survived (red); (**d**) cell counting at Days 13 to 15 of differentiation showed the very high purity of wild-type and Scn5a∆/+ cardiomyocytes. Scale bars: 50 μm. Error bars: S.E.M. EB, embryoid body.

*In vitro* differentiation of αPaG-RexNeo wild-type and Scn5a∆/+ iPS cells was performed using the hanging drop method for embryoid body (EB) generation [21] followed by a mass culture protocol (Figure 2a) [16]. EBs showed spontaneously beating areas at Days 10 to 12 of differentiation with weak GFP signals. At this stage, cardiomyocyte selection was started by puromycin application for one day, and single cells were re-plated on fibronectin-coated culture dishes. Longer selection at the EB stage was inefficient, because dissociation of older and more compact EBs with enhanced extracellular matrix failed, resulting in a low number of single cardiomyocytes. Single dissociated cardiomyocytes were spontaneously beating and weakly GFP-positive (Figure 2b).

Because cells without GFP expression or contractions were still present (Figure 2b, arrows), cultures were maintained under a low dose of puromycin selection, which led to further purification. Subsequently, the purity of cardiomyocytes was assessed by staining against cardiac α-actinin and cell nuclei (Figure 2c), and quantitative cell counting showed an almost pure population of cardiomyocytes (Figure 2d) from wild-type (92.8% ± 6.2%, *n* = 5) and Scn5a∆/+ iPS cells (87.7% ± 9.7%, *n* = 4).

### 3.3. Phenotyping of Purified LQTS 3-Specific Cardiomyocytes from Scn5a∆/+ iPS Cells

**Figure 3:**
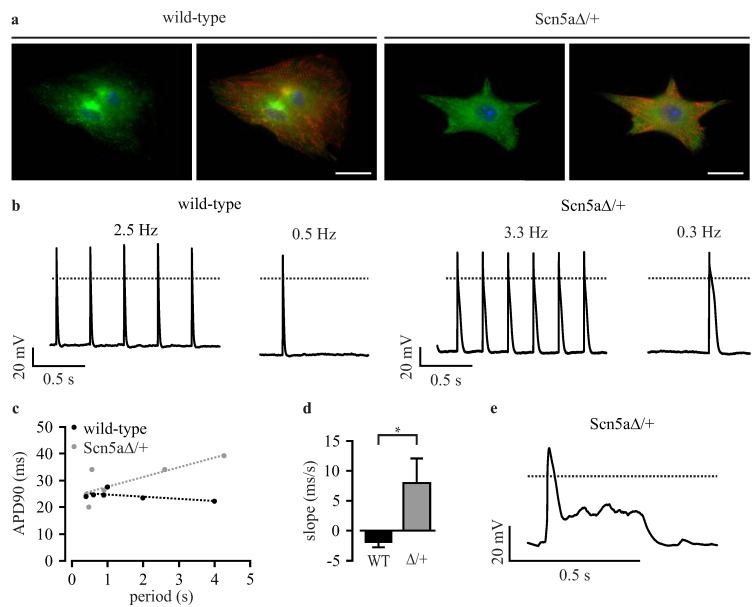
Patch clamp analysis of purified iPS-derived cardiomyocytes. (**a**) Cardiomyocytes from wild-type and Scn5a∆/+ iPS cells showed a similar cardiac sodium channel distribution (green) and sarcomeric α-actinin pattern (red); (**b**) representative examples of action potentials (APs) from purified wild-type and Scn5a∆/+ cardiomyocytes at high and low pacing frequencies; (**c**) relationship between action potential duration at 90% of repolarization (APD90) and pacing period from a representative wild-type and Scn5a∆/+ cardiomyocyte with the analysis of the slope by linear fit (dashed lines); (**d**) statistical analysis of the slope of APD90 to the pacing period relationship from individual wild-type (WT) and Scn5a∆/+ cardiomyocytes (∆/+); (**e**) typical long QT syndrome 3 (LQTS 3)-specific early afterdepolarizations (EADs) observed in a Scn5a∆/+ cardiomyocyte. Scale bar: 20 μm. Error bars: S.E.M. Dotted lines indicate 0 mV.

Purified cardiomyocytes from wild-type and Scn5a∆/+ iPS cells showed no obvious difference in cardiac sodium channel distribution or sarcomeric structure (Figure 3a). To exclude that the lentivirus integration, the non-clonal strategy or the purification affect the LQTS 3-specific phenotype we characterized purified cardiomyocytes by classical manual patch clamp techniques.

APs were evoked at various frequencies by current injection, and the frequency-dependent action potential duration at 90% repolarization (APD90) was analyzed. In Scn5a∆/+ cardiomyocytes, but not in wild-type cells, we found a prolongation of APD90 at lower heart rates (Figure 3b, Table 1), which did not reach statistical significance because of the high variability of APD90 between individual cells. The high variability was not due to the non-clonal purification approach, because it was similarly observed in non-purified cardiomyocytes from the original iPS cell clones [5]. To compensate for this variability, we performed a longitudinal analysis for each individual cell and determined the slope of the relationship between APD90 and basic cycle length (APD restitution) using a linear fit (examples shown in Figure 3c), as reported before [5,22]. This analysis showed almost no influence of cycle length on APD90 in purified wild-type cardiomyocytes yielding flat slopes of APD restitution (−1.85 ± 0.73 ms/s, *n* = 10, Figure 3d). In contrast, purified Scn5a∆/+ cardiomyocytes had a significant different positive slope (7.94 ± 4.05 ms/s, *n* = 18, Figure 3d) highlighting the prolongation of APD90 with a longer cycle length. This is the characteristic feature of LQTS 3 in patients [22] and is fully in line with previous reports on non-purified cardiomyocytes from Scn5a∆/+ iPS cells [5], as well as on cardiomyocytes from the ΔKPQ LQTS 3 mouse model [23]. Importantly, these slope values are almost identical to those obtained from the non-purified original iPS cell clones (wild-type: −2.92 ± 1.27 ms/s; Scn5aΔ/+: 9.08 ± 3.60 ms/s; see Table 2 in Malan* et al.* [5]). Moreover, we detected EADs in some purified Scn5a∆/+ cardiomyocytes (10.5%, *n* = 19, Figure 3e), but never in wild-type cells (0%, *n* = 10). Resting membrane potential, action potential amplitude and maximum upstroke velocity were not different between wild-type and Scn5a∆/+ cardiomyocytes (Table 1).

**Table 1:** Action potential parameters determined by manual and automated planar patch clamp analysis. RMP, resting membrane potential; APA, action potential amplitude; Vmax, maximum upstroke velocity; APD90, APD at 90% of repolarization; Slope, slope of the linear relationship between APD90 and the pacing period. Values are the means ± S.E.M.

Method	Manual Patch Clamp			Automated Planar Patch Clamp		
Genotype	Wild-Type	Scn5aΔ/+	*p*-Value	Wild-Type	Scn5aΔ/+	*p*-Value
RMP at 1 Hz	−77.4 ± 4.9	−74.7 ± 3.5	0.6589	−69.4 ± 6.4	−78.2 ± 3.0	0.2040
(mV)	*n* = 10	*n* = 17		*n* = 7	*n* = 9	
APA 1 Hz	106.2 ± 5.7	103.3 ± 5.7	0.7207	81.1 ± 13.2	85.8 ± 10.1	0.7746
(mV)	*n* = 10	*n* = 17		*n* = 7	*n* = 9	
V max at 1 Hz	93.7 ± 11.1	71.6 ± 9.6	0.1484	56.6 ± 15.5	52.6 ± 10.2	0.8248
(V/s)	*n* = 10	*n* = 17		*n* = 7	*n* = 9	
APD90 at 2 Hz	36.2 ± 3.5	39.5 ± 5.1	0.6004	78.0 ± 28.7	64.9 ± 17.1	0.7019
(ms)	*n* = 10	*n* = 14		*n* = 7	*n* = 7	
APD90 at 1 Hz	35.5 ± 3.3	45.8 ± 6.8	0.1913	70.1 ± 26.6	76.7 ± 20.4	0.8454
(ms)	*n* = 10	*n* = 15		*n* = 7	*n* = 9	
APD90 at 0.2 Hz (manual) or 0.5 Hz (automated) (ms)	39.7 ± 2.2	46.8 ± 6.7	0.1761	69.6 ± 24.3	91.5 ± 25.0	0.5440
	*n* = 5	*n* = 5		*n* = 7	*n* = 8	
Slope	−1.85 ± 0.73	7.94 ± 4.05	0.0287	−5.39 ± 4.82	4.13 ± 1.20	0.0494
(ms/s)	*n* = 10	*n* = 18		*n* = 7	*n* = 9	

Importantly, we did not find differences in action potential parameters between early and late passages of the non-clonal iPS cell clones (Table 2).

**Table 2:** Action potential parameters at early (P12–P18) and late (P19–P35) passages determined by manual patch clamp recordings (the abbreviations are as in Table 1).

Genotype	Wild-Type			Scn5aΔ/+		
Passage	Early Passage	Late Passage	*p*-Value	Early Passage	Late Passage	*p*-Value
RMP at 1 Hz	−71.8 ± 2.3	−68.3 ± 9.7	0.7061	−71.5 ± 7.3	−67.3 ± 5.6	0.6593
(mV)	*n* = 4	*n* = 3		*n* = 4	*n* = 7	
APA 1 Hz	105.5 ± 6.9	99.7 ± 16.5	0.7312	97.3 ± 9.0	91.3 ± 8.1	0.6513
(mV)	*n* = 4	*n* = 3		*n* = 4	*n* = 7	
V max at 1 Hz	98.8 ± 6.8	88.3 ± 31.7	0.7215	81.8 ± 15.6	59.6 ± 17.6	0.4230
(V/s)	*n* = 4	*n* = 3		*n* = 4	*n* = 7	
APD90 at 1 Hz	33.1 ± 3.6	27.7 ± 0.7	0.2657	49.1 ± 15.7	52.8 ± 11.3	0.8499
(ms)	*n* = 4	*n* = 3		*n* = 4	*n* = 7	
Slope	−1.10 ± 0.32	−1.10 ± 0.57	1.00	14.75 ± 13.12	8.94 ± 6.68	0.7106
(ms/s)	*n* = 4	*n* = 3		*n* = 4	*n* = 8	

### 3.4. Automated Electrophysiological Investigation and AP Measurements of Purified Cardiomyocytes with a Planar Patch Clamp System

In order to implement the use of purified wild-type and Scn5a∆/+ cardiomyocytes for automated screenings, we performed electrophysiological analysis with a planar patch clamp robot (Patchliner, Nanion Technologies). For this technique, freshly dissociated single cells in suspension are required. Therefore, a new and gentle dissociation procedure was used to minimize cell stress and to avoid partial digestion of ion channels, which are required for intact AP generation. Dissociation was facilitated by removal of Ca^2+^ and cooling of cells at 4 °C, which allowed subsequent dissociation with the very short application of Trypsin. After dissociation, cells were gently centrifuged and carefully resuspended in external solution. In order to let cardiomyocytes recover from the dissociation process, cells remained at least 2 h at room temperature before planar patch clamp experiments were performed. To verify the dissociation efficiency, single cells were counted, and the cell concentration was adjusted to 0.1 to 1 × 10^6^ cells/mL to ensure a good catch rate by the planar patch clamp robot. For planar patch clamp measurements, 20 μL containing 2000 to 20,000 purified cardiomyocytes, was automatically pipetted into each recoding unit of the planar patch clamp chip. Once a cell was caught, a negative pressure was automatically applied and a seal enhancer was injected to form a good GΩ*-*seal for stable recording without leaks.

**Figure 4:**
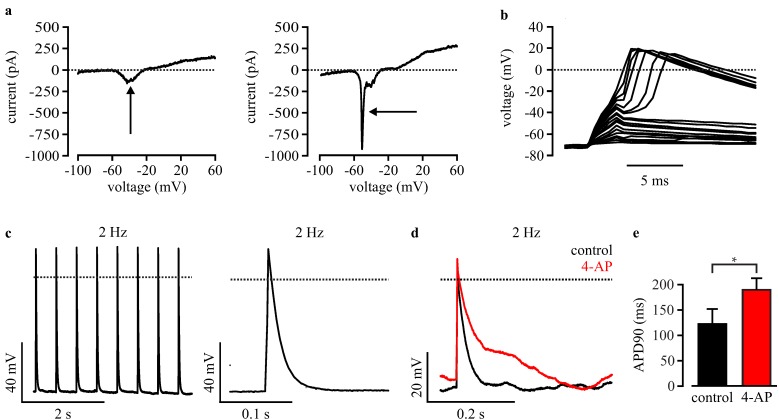
Automated planar patch clamp analysis of purified wild-type iPS-derived cardiomyocytes. (**a**) Examples of voltage ramps of an immature cardiomyocyte with Ca^2+^ current (left, arrow) and of a more mature cell with an additional fast Na^+^ current (right, arrow); (**b**) Representative membrane potential changes in response to stepwise increasing current pulses during the protocol for finding the AP threshold; (**c**) Example of automated AP recording at fixed pacing rate (**left**) with magnification (**right**); (**d**) representative APs before (black) and after blocking of K^+^ channels with automated application of 4-AP (red); (**e**) Statistical analysis of APD90 measured under control conditions and after 4-AP application. Error bars: S.E.M. Dotted lines indicate 0 mV or 0 pA.

To estimate the quality of recording and the differentiation stage of cardiomyocytes, depolarizing voltage ramps were applied in the voltage clamp mode. This allowed determination of the intact seal without major leak conductance, as well as the detection of typical inward and outward currents of voltage-dependent ion channels (Figure 4a). Voltage ramps were also used to classify cardiomyocytes in immature cells with a slow inward Ca^2+^ current peak (Figure 4a, left, arrow) and in more mature cells with an additional fast Na^+^ current component (Figure 4a, right, arrow). Because we wanted to characterize a disease based on a Na^+^ channel mutation, only cardiomyocytes with a clear Na^+^ current peak were subsequently used to record APs. APs were evoked by current injection in the current clamp mode (Figure 4c). To identify the minimal current required, a special protocol was executed by the PatchControlHT software (Nanion Technologies). Briefly, stepwise (100 pA steps) increasing 2 ms-long current stimuli were applied, and the voltage responses were analyzed (Figure 4b). As soon as the resulting amplitude was >30 mV above the resting membrane potential, the applied current was defined as the threshold-current and 80 pA was added for safety.

To determine if AP recordings with a planar patch clamp system are useful to investigate LQTSs that mainly affects cardiac repolarization, we inhibited the repolarizing K^+^ channels by automated application of 4-aminopyridine and measured the effect on APD90. As expected, we found AP prolongation in purified wild-type cardiomyocytes from 120.1 ± 30.5 ms to 188.9 ± 24.0 ms (*n* = 4, AP evoked at 2 Hz, Figure 4d,e).

### 3.5. Automated Phenotypic Characterization of LQTS 3-Specific Purified Cardiomyocytes from Scn5a∆/+ iPS Cells with a Planar Patch Clamp Robot

**Figure 5:**
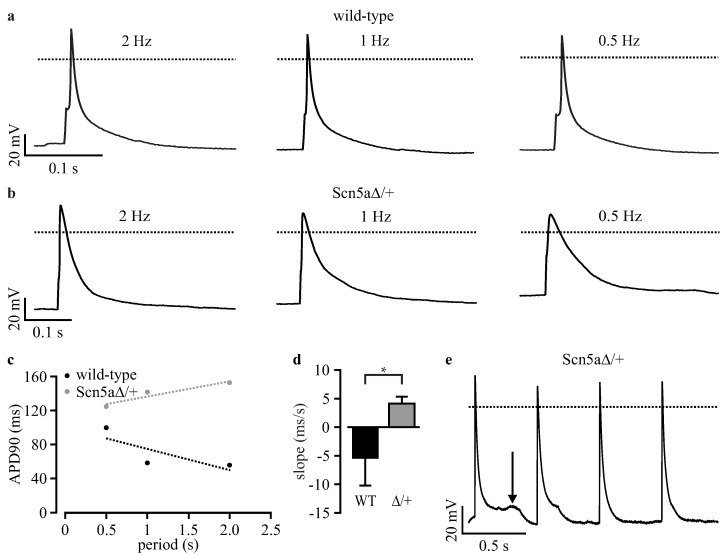
Automated characterization of LQTS 3-specific cardiomyocytes by planar patch clamp. (**a**,**b**) Representative traces of APs from wild-type (**a**) and Scn5a∆/+ (**b**) cardiomyocytes at high and low pacing frequencies; (**c**) relationship between APD90 and the pacing period from a representative wild-type and a Scn5a∆/+ cardiomyocyte with analysis of the slope by linear fit (dashed lines); (**d**) statistical analysis of the slope of APD90 to the pacing period relationship from individual wild-type (WT) and Scn5a∆/+ cardiomyocytes (∆/+); (**e**) typical LQTS 3-specific EADs observed in a Scn5a∆/+ cardiomyocyte (arrow). Error bars: S.E.M. Dotted lines indicate 0 mV.

To proof the feasibility to characterize LQTSs with automated electrophysiological analysis, we recorded APs from purified cardiomyocytes using the planar patch clamp system. Frequency dependence was determined with APs elicited at 2, 1 and 0.5 Hz pacing frequencies using the automatically determined current threshold (see the above Section 3.4). Similar to the results from manual patch clamp recordings (Figure 3), we found prolonged APs at low heart rates in Scn5a∆/+ cardiomyocytes, but not in wild-type cells (Figure 5a,b). Furthermore, the longitudinal analysis of APD restitution in individual cells (examples shown in Figure 5c) showed a positive slope in Scn5a∆/+ cardiomyocytes (4.13 ± 1.20 ms/s, *n* = 9) and a significant different negative slope in wild-type cells (−5.39 ± 4.82 ms/s, *n* = 7, Figure 5d, Table 1). Finally, we observed EADs in 30% of purified Scn5a∆/+ cardiomyocytes (Figure 4e, *n* = 10), but none in wild-type cells (*n* = 7).

### 3.6. Analysis of Field Potentials from Purified iPS Cell-Derived Cardiomyocytes with Microelectrode Arrays

**Figure 6:**
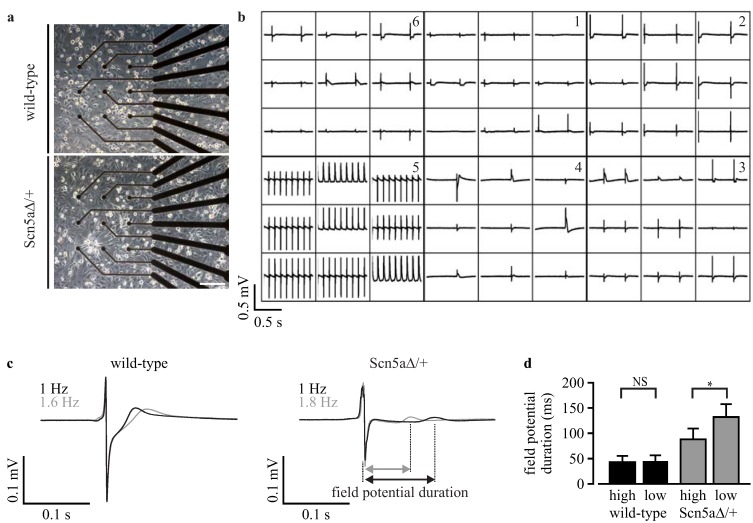
Field potential analysis with microelectrode arrays. (**a**) Image of the recording electrodes of a six-well microelectrode array with a monolayer of purified cardiomyocytes; (**b**) overview of field potential recordings from three wells with purified wild-type (**bottom**) and three wells with Scn5a∆/+ cardiomyocytes (**top**); (**c**) examples of averaged field potentials with slow beating at 22 °C (black) and faster beating at 37 °C (grey) from purified wild-type and Scn5a∆/+ cardiomyocytes. The analysis of the field potential duration is shown in the Scn5a∆/+ recording; (**d**) statistical analysis of the field potential durations at low and high spontaneous beating frequencies from wild-type and Scn5a∆/+ cardiomyocytes. Scale bar: 200 μm. Error bars: S.E.M. NS, not significant.

The duration of APs can not only be determined by patch clamp analysis, but can also be estimated indirectly from extracellular field potential recordings with microelectrode arrays, because of the good correlation of field potential duration to APD [24]. To prove the functionality of this technology for the characterization of LQTS 3, we plated purified cardiomyocytes obtained from wild-type and Scn5a∆/+ iPS cells on six-well microelectrode arrays on which they formed a monolayer of synchronously beating cells (Figure 6a). This allowed recordings of field potentials from nine electrodes in six individual wells (example recording in Figure 6b). To determine frequency-dependent field potential duration, measurements were performed at 22 °C and at 37 °C, which accelerated the spontaneous beating frequency.

Field potential duration was analyzed after trigger-based averaging over 50 s and calculation of the mean field potential from all nine electrodes (for details, see Section 2.7), resulting in one averaged field potential for each well (examples in Figure 6c). Scn5a∆/+ cardiomyocytes showed a significantly (*p* = 0.011) longer field potential duration at low frequencies (132.4 ± 25.2 ms, *n* = 3) compared to high frequencies (88.0 ± 22.6 ms, *n* = 3, Figure 6d). Importantly, such a frequency-dependent effect was not observed in wild-type cardiomyocytes (low frequency: 45.5 ± 9.5 ms, *n* = 3; high frequency: 42.1 ± 10.5 ms, *n* = 3; *p* = 0.78). Thus, also field potential analysis with a microelectrode array showed the disease-specific frequency dependence of prolonged AP durations in purified Scn5a∆/+ cardiomyocytes.

## 4. Discussion

In this study, we present a novel, simple and fast lentiviral strategy for the purification of cardiomyocytes from iPS cells and show the feasibility of using these cells for automated electrophysiological investigations. The adverse effects of the random lentivirus integration, the non-clonal cell selection and the antibiotic purification on the pluripotency of iPS cells or the electrophysiological characteristics of cardiomyocytes were not detected. Importantly, purified cardiomyocytes had fast depolarizing Na^+^ currents, AP generation and intact repolarization by K^+^ currents and, therefore, were well suited to investigate LQTS in which these parameters are affected. We have proven this by showing the intact electrophysiological phenotype of purified LQTS 3-specific cardiomyocytes from previously published Scn5a∆/+ iPS cells [5]. Furthermore, we have characterized purified cells with the automated planar patch clamp recordings and scalable microelectrode array analysis, which highlights the usefulness of these technologies for drug screening. Following, we discuss the achievements so far and the hurdles to overcome for large-scale purification and electrophysiological screening of cardiomyocytes.

### 4.1. Lentiviral Non-Clonal Gene Transfer Strategy

We have chosen cardiac-specific expression of an antibiotic resistance gene in order to kill all non-cardiomyocytes by antibiotic application. In contrast to low throughput single-cell sorting of labeled cardiomyocytes [14,15], this strategy enables the large-scale purification of cells. Because transfection of plasmid with common chemical, electroporation or lipofection methods suffers from poor efficiency in undifferentiated iPS cells, viral gene transfer methods are suitable alternatives [25,26]. We have used a lentivirus strategy that allows not only high efficient gene transfer, but also stable integration into the genome [25]. In addition to the cardiac-specific antibiotic resistance, we have employed a neomycin resistance gene under the control of the stem cell-specific promoter, Rex-1 [20], which was shown before to be useful for the selection of embryonic stem cell clones [17]. Thus, neomycin treatment allowed the selection of undifferentiated cells with stable lentivirus integration and without silencing or adverse positional effects of the surrounding host chromatin. One further advantage of using Rex-1-neomycin is that the continuous selection pressure at undifferentiated stages prevents iPS cell differentiation or lentiviral silencing at higher passages.

Usually, after classical or viral gene transfer into pluripotent stem cells, several single-cell clones are picked, propagated and characterized individually [16], a very time-consuming procedure and, therefore, an expensive task. In contrast to previous work, we decided to pool all iPS cells that survived the neomycin selection and generated one non-clonal iPS cell line for each genotype. This strategy harbors the risk that a single iPS cell clone with enhanced proliferation by lentivirus-induced mutations or chromosomal aberrations could overgrow the mixed population. However, the intact stem-cell morphology, the expression of stem cell markers, the normal proliferation of the mixed clones and the high similarity of all electrophysiological parameters in cardiomyocytes from early and late passages of the non-clonal iPS cell lines suggest no adverse effects of this strategy. Importantly, the phenotypical fingerprint of LQTS 3 (APD prolongation at slow rates) was only observed in Scn5a∆/+ cardiomyocytes, both at early and late passages. Furthermore, the slope values of cardiomyocytes from the non-clonal iPS cells were almost identical to those from the original iPS cell clones [5].

The novel possibility to work with non-clonal iPS cells is also supported by a previous report on the successful generation of iPS cell clones in bulk culture without clone picking, which did not reveal differences with clonal selected iPS cell lines regarding pluripotency, gene expression profiles or differentiation potential [27]. Because a non-clonal strategy avoids manual clone picking and could be applied in 96-well or scalable formats, it enables the parallel generation and genetic modification of iPS cell lines from different patients at once. This would allow the purification of cardiomyocytes from many different patients for parallel and comparative electrophysiological screening.

The non-clonal lentiviral cardiomyocyte purification strategy might also have limitations and variations in efficacy because of uncontrolled variations in copy numbers and integration sites between iPS cells. High concentrations of neomycin could be used for selecting clones with the highest copy numbers, and this should be investigated in the future. Because lentiviruses have the tendency to integrate into euchromatin [28], infection at the stem cell level could lead to clones that are neomycin resistant at undifferentiated stages, but encounter lentiviral silencing upon differentiation and, therefore, fail to express puromycin for cardiomyocyte purification. Furthermore, the random integration of lentiviruses could cause insertional mutagenesis; however, this seems not to be frequent, because they tend to integrate away from promoters [29].

Recently, metabolic selection by the cultivation of stem-cell-derived cells in glucose-depleted medium containing only lactate as the energy source was described to be an efficient non-genetic method for the purification of cardiomyocytes [30]. Although the authors report a purity of 99% cardiomyocytes, this method seems to be highly dependent on the cell line used. In fact, although we have extensively tried to reproduce these purity values, we only obtained 45%–80% cardiomyocytes from mouse embryonic stem and human iPS cell lines using identical metabolic selection procedures [31].

### 4.2. Choice of a Cardiac-Specific Promoter

For cardiac-specific expression of the puromycin resistance gene, we have used the α-MHC promoter, which was shown to enable high efficient purification of cardiomyocytes from mouse and human iPS and embryonic stem cells [16,17,32,33]. Because of the size limitation for gene transfer using lentivirus (~9–10 kb between LTRs [27]), we had to use a short version (~1.7 kb) of the 3′ end of the classical 6.5 kb-long α-MHC promoter. Although this fragment contained important gene expression regulatory elements (TATA box, MEF-1 MEF-2 and Nkx2.5 binding sites) [34], it is likely that unidentified enhancing elements were not present explaining the weak GFP expression. Nevertheless, purification of cardiomyocytes was unharmed, indicating sufficient expression of the puromycin resistance gene. This indicates a lower threshold for puromycin resistance than for GFP fluorescence, because the use of a 2A self-cleaving peptide should result in equimolar expression of both proteins [18].

In the future, the use of other promoters should be considered. Although the α-MHC promoter is labeling mature cardiomyocytes in mice, β-MHC is the predominant isoform in the human ventricle, and α-MHC is a marker rather for atrial or failing human cardiomyocytes [35]. Therefore, mature cardiomyocytes from human iPS cells should be selected with the β-MHC promoter. Furthermore, the choice of other subtype-specific promoters could be very useful to obtain the cardiomyocyte population of interest. For instance, LQTS could be best investigated in ventricular cardiomyocytes that have long AP durations and could be selected using the MLC2v promoter. Moreover, mutations inducing atrial fibrillation might be better investigated with atrial cell selection by the MLC2a promoter, and for studying inherited sick sinus syndromes, pacemaker cells could be purified with sinus node-specific HCN or Tbx promoters.

### 4.3. Automatable and Scalable Electrophysiological Screening

The use of screening procedures to analyze APs of iPS cell-derived cardiomyocytes is particularly important to identify drugs that induce LQTS or to screen compounds that could treat inherited LQTS. For the systematic screening of many compounds, the classical manual patch clamp is not suitable, and automated and scalable systems are mandatory. For instance, the planar patch clamp technique [12] or the microelectrode array system [24,36] allow the acquisition of more data points per day (planar patch clamp: 200–1000; microelectrode array: 500) than the conventional patch clamp (50 data points/day) [36].

The planar patch clamp system that we have used in this study allows the automated recording of up to eight cells in parallel, as well as the automated application of several compounds. Because cells must be measured in suspension, very gentle dissociation methods have to be further optimized to avoid digestion of transmembrane ion channels.

We found that most action potential parameters were similar between manual and planar patch clamp recordings; however, APD90 tends to be longer (statistically not significant) in the latter (Table 1). We speculate that when using the automated planar patch clamp method, the dissociation procedure or the suction process onto the small holes of the borosilicate glass chips could kill smaller atrial or pacemaker cells with shorter APD or might favor larger ventricular cells with longer APD. However, although absolute APD values seems to vary with the method, the phenotypical fingerprint of LQTS 3-specific cardiomyocytes (positive slopes in the longitudinal regression analysis) can be similarly detected with both patch clamp methods (Table 1).

Similar to the conventional patch clamp, also during automated planar patch clamp analysis, the intracellular milieu is dialyzed against the internal solution, which leads to the wash out of important intracellular components and, therefore, reduces the stability of long-term recordings. This limits the duration of electrophysiological recording of one cell and, therefore, also the number of different compounds and dosages. Thus, this technology seems to be not suited for real high throughput analysis of several thousands of compounds.

Although, here, we only performed six recordings on a microelectrode array in parallel, scalable and automatable systems were developed (QT screen Multi Channel Systems) for parallel field potential recording and compound testing on 96 channels. In contrast to conventional microelectrode measurements (500 data points/day), such systems allow the recoding of 6000 data points/day [36]. One remaining challenge is the almost impossible electrical stimulation of cardiomyocytes on microelectrode arrays for standardized recordings and to determine frequency-dependent effects. This could be solved by using optogenetic technology, which was shown to be effective for the stimulation of purified cardiomyocytes on microelectrode arrays [37].

## 5. Conclusions

The herein reported non-clonal lentiviral strategy for the purification of cardiomyocytes from iPS cells is simple, fast and cheap and could be applied to large numbers of different iPS cell lines at once. In contrast to the picking of classically-transfected iPS cell clones, this strategy would allow the parallel purification of cardiomyocytes from many different patients for comparative electrophysiological analysis. Because the disease-specific phenotype of purified iPS cell-derived cardiomyocytes was retained and could be analyzed with automated planar patch clamp and scalable microelectrode array technologies, these assay systems will be useful for patient-specific drug screening in the future.
